# A Bibliometric Analysis of the 50 Most Commonly Cited Studies of the Direct Anterior Approach in Total Hip Arthroplasty

**DOI:** 10.1155/2022/1974090

**Published:** 2022-06-17

**Authors:** Ramakanth R. Yakkanti, Anil Sedani, Dylan N. Greif, Rukmini Yakkanti, Dustin H. Massel, Victor H. Hernandez

**Affiliations:** ^1^University of Miami Miller School of Medicine, Department of Orthopaedics, Miami, FL, USA; ^2^University of Miami Miller School of Medicine, Miami, FL, USA; ^3^University of Miami Miller School of Medicine, University of Miami Sport Medicine Institute, Coral Gables, FL, USA; ^4^Kentucky College of Osteopathic Medicine, Pikeville, KY, USA

## Abstract

**Introduction:**

The direct anterior approach (DAA) has garnered a lot of attention and surgical popularity for total hip arthroplasty in recent years. Some of the postulated advantages for the increase in popularity of this approach include better pain control and earlier recovery in the immediate postoperative period. The amount of literature available on this topic has rapidly increased over the last 10 years requiring the need for an updated guide to best navigate the topic.

**Methods:**

The ISI Web of Knowledge database was used to search for research articles regarding the DAA surgical technique. The Boolean operative that yielded the largest search results was ([direct anterior approach hip] OR [anterior hip] OR [anterior hip arthroplasty] NOT [spine]).

**Results:**

92% of the highest cited articles on the DAA were published within the past two decades. One author, Keggi K, published 4 or more articles, with the highest citation average (110.5 citations). Recent articles were more strongly correlated with higher citation counts (*R*^2^ = 0.21 *v*. 0.19).

**Conclusion:**

This review clearly outlines the increasing trend in the most influential publications regarding DAA being published in the past two decades. This review allows interested surgeons to understand the historic literature pertaining to this topic. This review can assist future researchers in identifying trends in UKA as well as help clinicians navigate this body of literature.

## 1. Introduction

Surgical techniques and approaches in joint replacements are evolving. Recent trends in literature demonstrate that the direct anterior approach (DAA) has garnered popularity as a surgical treatment option for total hip arthroplasty. The increase in popularity of the DAA may, in part, be the result of the postulated advantages; the DAA affords improved postoperative pain control, and as a result of the muscle-sparing approach, and an earlier and faster recovery in the immediate postoperative period [1]. While there is an increased use of this technique, there remains an ongoing discussion regarding the efficacy and complications of the approach. The amount of literature available on this topic has rapidly increased over the last 10 years; there were 653 articles cited in PubMed since the start of 2019 regarding the DAA, further requiring the need for an updated guide to best navigate the topic.

The evaluation of published literature was traditionally driven by review articles and surveys; however, bibliometric analyses have become a very important and standard tool over the past few decades. Such an analysis is made to explore the qualities and characteristics of the many published articles regarding a specific topic. The first of this kind was published in the Journal of American Medical Association (JAMA) in 1987 [2]. Bibliometric articles provide a cross-sectional analysis of a field of research and may assist in the identification of the most impactful publications in the present literature. As the number of published articles or trends increases over time, bibliometric analyses assist in the investigation and evaluation of the level of scientific significance of the research available. In the current review, published literatures examining the anterior approach for total hip arthroplasty were analyzed.

There are previous bibliometric reviews regarding total hip arthroplasty [3, 4]. Zhang et al. and Miso et al. discussed total hip arthroplasty as a whole and identified important original articles in all major topics of total hip arthroplasty. As the interest in DAA grows, a bibliometric analysis is invaluable to the orthopaedic community to help navigate the growing body of literature and aid surgeons in identifying important and influential literature on DAA. With statistical analysis of the most frequently cited articles, the significance of the available literature can be correlated with some degree, though with certain limitations. To the best of the authors' knowledge, the current review is the first to apply bibliometric analysis to evaluate the quality of literature available for the direct anterior approach for total hip arthroplasty. The aim of this study is to identify the top 50 most cited articles related to the focused topic of the DAA. Furthermore, the purpose of this study is to offer a tool to orthopaedic surgeons to efficiently navigate the ever-expanding literature on the surgical technique and perioperative management of patients receiving total hip arthroplasties with the DAA.

## 2. Methods

The Institute of Scientific Information (ISI) Web of Knowledge database was used to search for research literature discussing the anterior hip approach surgical technique. A literature query was conducted in January 2020 with multiple Boolean operative combinations by two independent reviewers (RY and DG). The Boolean operative that yielded the largest search results was ([direct anterior approach hip] OR [anterior hip] OR [anterior hip arthroplasty] NOT [spine]).

Initial query was not limited by any language of publication, date range, journal, or article type. Search results were then refined to include only peer-reviewed original articles, review papers, or editorials and were then ordered by a total number of citations. Articles were not excluded for level of evidence, number of citations, language of publication, or date of publication.

Articles were then evaluated by two independent reviewers (RY and AS) for their relevance to orthopaedics. Data extracted from these articles included: manuscript title, first author, last author, total citation count, month and year of publication, clinical category, citation density since the date of publication, current citation rate since 2013, journal name, country of origin, and level of evidence (LOE). LOE was determined via the Canadian Task Force Periodic Health Examination and the Center for Evidence-based Medicine [5].

Articles were then categorized by the following themes based on the clinical research question proposed by the authors: (1) Surgical Technique and Outcomes, (2) Complications, (3) Imaging, and (4) Anatomy.

### 2.1. Statistics

The Shapiro–Wilk test was used to test the distribution of individual variables for normality. Normally distributed data are presented with means and standard deviations. One-way ANOVA (analysis of variance) was utilized to assess differences among normally distributed data. The Kruskal–Wallis test was used for skewed data. The Spearman rank was utilized to test for correlations among variables. Statistical significance was set at *p* < 0.05. Microsoft Excel version 16.33 was utilized for statistical analysis.

## 3. Results

The initial search yielded 1,032 articles, and the top 50 most cited articles were included for analysis (Table 1). All included publications were published between 1980 and 2017. The highest-ranked paper was cited 287 times, while the lowest-ranked paper was cited 59 times (Table 1). The average number of citations per publication was 75, while the average citation density since the year of publication was 9.3. The average citation rate since 2013 was 0.962.

**Figure 1:**
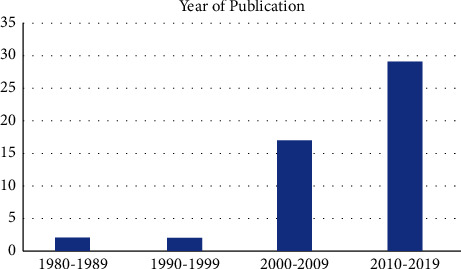
Journal articles published by decade.

The first publication on our list was in 1980 by Terry and Keggi [6]. Most papers were published in the past decade, 2010–2019 (*n* = 29, 58%), followed by 2000–2009 (*n* = 17, 34%) (Figure 1). The most prolific single year of publication was 2009, with 9 publications. The 4^th^-ranked paper had the highest citation density (24.47) and was published in 2013, while the top-cited paper was published in 2005 with 287 citations. The 40^th^-ranked paper had the lowest citation density (0.14) since the year of publication (1985).

**Figure 2:**
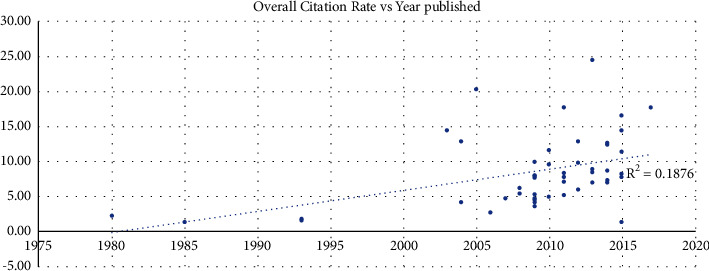
Overall citation density vs year published.

**Figure 3:**
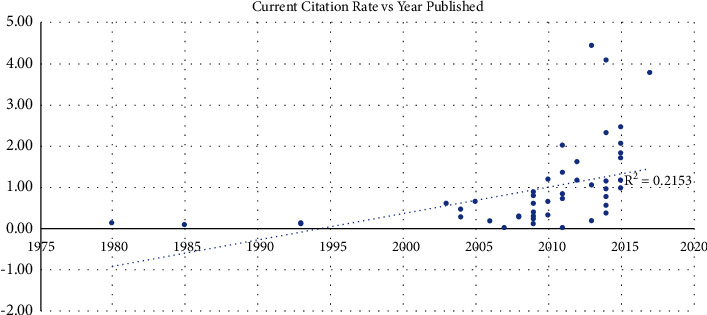
Current citation density vs year published.

A positive correlation *R* = 0.43 (*p*=0.002) was seen between the year published and citation density, while a regression analysis found an *R*^2^ value of 0.19 (Figure 2). When evaluating the current citation rate since 2013, a positive correlation was also observed (*R* = 0.46, R^2 = 0.21, *p* < 0.001) (Figure 3).

**Figure 4:**
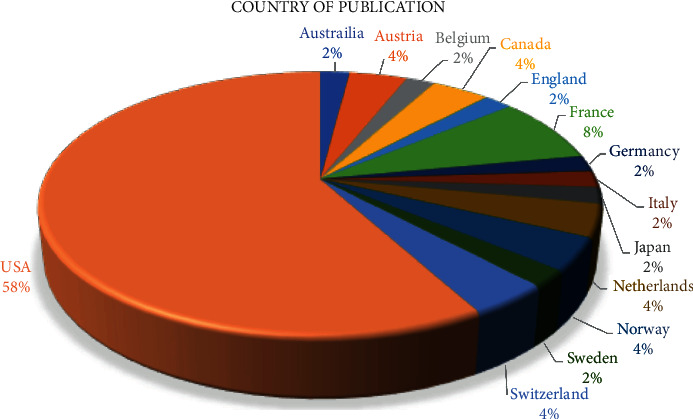
Country of publication.

The country with the greatest contribution to publications on DAA was the United States (*n* = 29, 58%), followed by France (*n* = 4, 8%) (Figure 4). A total of 14 countries contributed to the growing body of literature.

**Figure 5:**
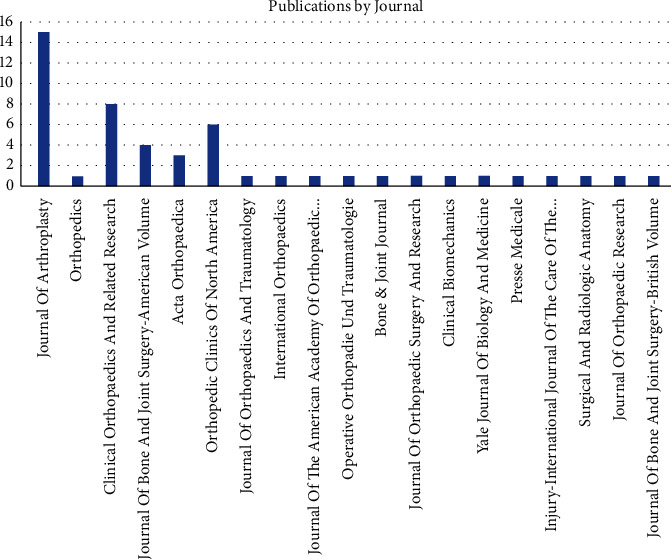
Publication by journal.

Among the 19 journals with published literature, the *Journal of Arthroplasty* published 15 articles, contributing to 30% of the top 50 most cited, followed by *Clinical Orthopaedics and Related Research* with 8 articles and 16% of the top 50 most cited (Figure 5).

**Figure 6:**
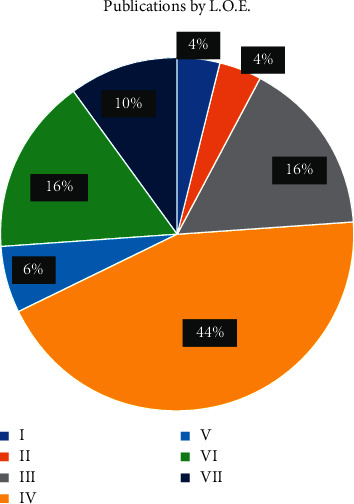
Citation per LOE.

When stratifying articles by topics discussed, the majority of articles focused on Surgical Technique/Outcomes (*n* = 29, 58%), followed by Complications (*n* = 18, 36%) (Figure 6). The remaining publications were related to Anatomy (*n* = 2, 4%) and Imaging (*n* = 1, 2%).

**Figure 7:**
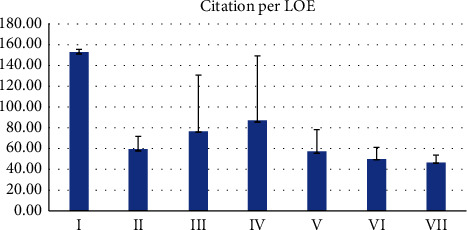
Mean citation per LOE.

**Figure 8:**
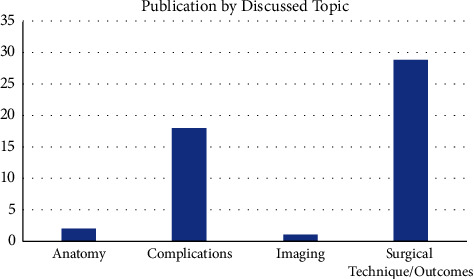
Publication by topic discussed.

The majority of papers were published with LOE of IV (*n* = 22, 44%), while only 2 papers were in level I (Figure 7). Articles published with LOE I had the highest citation rate (Mean = 152.5), although no significant difference in the total number of citations based on LOE was observed (*p*=0.12) (Figure 8).

One author, Keggi K, published 4 or more articles, with the highest citation average (110.5 citations). Seven authors had 2 publications (Kennon R.; Taunton M.; Christensen C.; Jacobs C.; Rathod P.; Rodriguez J.; and Leunig M.).

## 4. Discussion

Historically there are multiple surgical approaches for total hip arthroplasty. Three specific approaches are commonly used, including the direct anterior approach, the lateral approach, and the posterior approach [7, 8]. The popularity of DAA has increased recently [9], and it is demonstrated in our study that more recent articles are strongly correlated with higher citations counts (*R*^2^ = 0.21 *v*. 0.19). The DAA relies on an intermuscular plane for the deep dissection, limiting the damage to the muscles when compared to the posterior or lateral approach, resulting in improved postoperative pain and faster recovery [10]. With increased interest from surgeons and patients, the amount of research evaluating the DAA has greatly increased. Compared to prior literature on the more well-established approaches, many authors believe there is a lack of substantial published literature to fully evaluate the advantages and complication rates of the DAA [11]. In using Web of Science's search aggregation of over a dozen international research indexes, we were able to adequately capture the breadth of the evolving literature regarding DAA [12].

This review clearly outlines the increasing trend in the most influential publications regarding DAA being published in the past two decades. 92% of the highest cited articles on the DAA were published within the past two decades, further emphasizing the recent boon in the literature and practice. With this recent trend towards increased publications on the DAA, it is important to evaluate the available literature to identify the articles which have made the greatest impact (Figures 1 and 2). Given the stronger correlation of the current citation rate (since 2013) compared to the overall rate, the analysis reveals a more recent uptrend in the literature (Figures 2 and 3). With the top 50 most cited articles provided in the article, clinicians and patients are provided a tool to allow a deep dive into the most current literature regarding the DAA. Furthermore, clinicians are able to quickly identify the most impactful articles to investigate. Furthermore, in a 2018 American Association of Hip and Knee Surgeons (AAHKS) Survey at their annual meeting, they found that 40% of surgeons are using the DAA in THA as part of their practice, which is up from less than 10% in 2009. The recent trend in preference is mirrored in the literature with the surge of papers showering the literature.

The oldest article included in our analysis was published in 1980, which is not much younger than the oldest paper in our initial query on DAA, which was published in 1975. There is documentation of the Hueter anterior approach for femoral head arthroplasty performed by Judet in 1947, the approach has been modified to allow improved exposure of the acetabulum and femoral shaft [13, 14]. There is a clear trend towards more impactful and increased total number of publications by decade (Figures 1 and 2). Although the DAA was first described in the 1950s, few articles have been published in the following 5 decades. The data presented in the current review suggest a progressive interest in the DAA, especially in the last 2 decades (Figure 1).

The top three most cited articles described the technique utilized for a single-incision direct anterior approach for THA and the authors' early experience. The most cited article on the DAA is “Single-incision anterior approach for total hip arthroplasty on an orthopaedic table” by Matta et al. [15]. This was one of the first articles to report long-term results in a large cohort of patients who underwent a single-incision anterior approach for THA. Matta et al. demonstrated a low rate of dislocation at 0.61%, in part due to the muscle-sparing nature of this approach [15]. This article was influential in raising awareness about the DAA, its benefits compared to the older well-established approaches, and remains the most impactful article covering this topic.

The second most cited article is “Total hip arthroplasty through a minimally invasive anterior surgical approach” by Kennon and Keggi et al. (Table 1). Published in 2003, the authors report favorable postoperative outcomes following over 2000 procedures using the DAA. However, the popularity of the DAA and the impact of the Kennon et al. article did not become apparent until a decade later (Figure 2) [16]. The top three most cited articles were published between 2003 and 2005. The three articles most commonly cited likely contributed to increased interest in the DAA in the following decades [15–17]. Two out of the top three articles, “Single-incision anterior approach for total hip arthroplasty on an orthopaedic table” by Matta et al. and “Mini-incision anterior approach does not increase dislocation rate: a study of 1037 total hip replacements” by Siguier et al. (numbers 1 and 3, respectively, on our list) were also included in a recent bibliometric analysis about the most 100 influential articles in THA (Table 1). The paper by Matta et al. was number 50 on the list and the paper by Siguier et al. was 99 [3]. The bibliometric analysis about THA describes that the most influential articles primarily discussed the topics of postoperative thromboembolism and surgical methods and materials [3]. As DAA falls under the category of surgical methods and it is relatively recent in adoption in the time frame of THA when compared to some of the other more established approaches, it seems appropriate that the most influential article regarding DAA is listed as number 50 in the bibliometric analysis about THA. We predict that the impact of these articles regarding DAA will continue to increase in the upcoming decade, considering that interest and adoption of the DAA continues to rise.

The citation density is higher in papers written in the past decade (Figures 1 and 2). The paper with the highest citation density is “Prospective randomized study of direct anterior vs postero-lateral approach for total hip arthroplasty.” Barrett et al. published a randomized controlled trial comparing the posterolateral approach and the DAA. Although the posterolateral approach was considered the gold standard at the time of publication, the authors conclude the DAA results in reduced pain and early mobility in the immediate postoperative period. Although prior literature had published similar findings, this article was instrumental in providing level I evidence, to support the results of prior publications with more certainty [18]. Only one additional article in the top 50 most cited published level 1 evidence on the DAA, “Comparison of minimally invasive direct anterior versus posterior total hip arthroplasty based on inflammation and muscle damage markers” [19]. These two articles ranked as numbers 4 and 5 most cited in the current bibliometric analysis (Figures 7 and 8).

The USA had the highest number of articles and produced 58% of the most influential articles, followed by France producing 8%. The USA published 24% more articles than all of Europe combined (34%) (Figure 4). The greatest number of articles were published in the *Journal of Arthroplasty*, followed by *Clinical Orthopaedics and Related research*. The research is concentrated in the *Journal of Arthroplasty* likely because fellowship-trained THA surgeons are likely the first to adopt the DAA when compared to generalists who also perform THA surgery. This early adoption by fellowship-trained surgeons is likely due to the steep learning curve that has been associated with the implementation of this approach in practice [20, 21].

The majority of articles focused on Surgical Technique/Outcomes (58%), with Complications accounting for 36% of articles (Figure 8). This makes intuitive sense, in that arthroplasty surgeons may be most interested in outcomes and complications when implementing new techniques compared to the gold standard and other more established approaches.

Given the delay in citation counts of the most recent studies, which may over time become more highly cited, bibliometric analyses may miss early trends, highlighting both a limitation and a necessity of newer bibliometric analyses. Bibliometric analyses provide a retroactive view in to the literature but are limited in the ability to draw conclusions. Additionally, there have been limited data assessing the correlativity between citation count and strength of the article. The literature on bibliometric analyses highlights its limitations in capturing various nuances of citation counts, but offers a guide to navigate the literature on various topics [22].

## 5. Conclusion

The present article identifies the top 50 most cited articles in DAA for THA. With the increased trend in literature over the last 2 decades, it is important for surgeons performing THA to be familiar with the most influential literature on the DAA (Figures 1 and 2). The present analysis demonstrates the investigation priority lies in the postoperative outcomes and complications in comparison to well-established approaches. There is a paucity of high-quality evidence. Only two level I studies exist, while the majority of published literature are of level 4 evidence. We encourage researchers of the DAA to contribute more higher level of evidence studies. This review can assist future researchers and clinicians to identify the most influential trends in DAA for THA and allow more efficient application of the DAA within their practice and research.

## Figures and Tables

**Table 1:** Top 50 most cited articles regarding DAA.

Rank	Article	Total citations	Citations/year of publication until February 2020	Citations since 2013
1	Matta J. M., Shahrdar C., Ferguson T. Single-incision anterior approach for total hip arthroplasty on an orthopaedic table. Clin Orthop Relat Res. 2005; 441: 115–24.	287	20.26	9
2	Kennon R. E., Keggi J. M., Wetmore R. S., Zatorski L. E., Huo M. H., Keggi K. J. Total hip arthroplasty through a minimally invasive anterior surgical approach. J Bone Joint Surg Am. 2003; 85-A Suppl 4: 39–48.	244	14.35	10
3	Siguier T., Siguier M., Brumpt B. Mini-incision anterior approach does not increase dislocation rate: a study of 1037 total hip replacements. Clin Orthop Relat Res. 2004; (426):164–73.	198	12.84	4
4	Barrett W. P., Turner S. E., Leopold J. P. Prospective randomized study of direct anterior vs postero-lateral approach for total hip arthroplasty. J Arthroplasty. 2013; 28 (9):1634–8.	155	24.47	28
5	Bergin P. F., Doppelt J. D., Kephart C. J., et al. Comparison of minimally invasive direct anterior versus posterior total hip arthroplasty based on inflammation and muscle damage markers. J Bone Joint Surg Am. 2011; 93 (15):1392–8.	150	17.65	17
6	Jewett B. A., Collis D. K. High complication rate with anterior total hip arthroplasties on a fracture table. Clin Orthop Relat Res. 2011; 469 (2): 503–7.	115	11.60	3
7	Woolson S. T., Pouliot M. A., Huddleston J. I. Primary total hip arthroplasty using an anterior approach and a fracture table: short-term results from a community hospital. J Arthroplasty. 2009; 24 (7): 999–1005.	102	9.87	2
8	Spaans A. J., Van Den Hout J. A., Bolder S. B. High complication rate in the early experience of minimally invasive total hip arthroplasty by the direct anterior approach. Acta Orthop. 2012; 83 (4): 342–6.	96	12.80	12
9	Goulding K., Beaulé P. E., Kim P. R., Fazekas A. Incidence of lateral femoral cutaneous nerve neuropraxia after anterior approach hip arthroplasty. Clin Orthop Relat Res. 2010; 468 (9): 2397–404.	90	9.56	6
10	Alecci V., Valente M., Crucil M., Minerva M., Pellegrino C. M., Sabbadini D. D. Comparison of primary total hip replacements performed with a direct anterior approach versus the standard lateral approach: perioperative findings. J Orthop Traumatol. 2011; 12 (3): 123–9.	88	7.09	0
11	Berend K. R., Lombardi A. V., Seng B. E., Adams J. B. Enhanced early outcomes with the anterior supine intermuscular approach in primary total hip arthroplasty. J Bone Joint Surg Am. 2009; 91 Suppl 6: 107–20.	87	7.91	4
12	Light T. R., Keggi K. J. Anterior approach to hip arthroplasty. Clin Orthop Relat Res. 1980; (152): 255–60.	87	2.21	4
13	Bhandari M., Matta J. M., Dodgin D., et al. Outcomes following the single-incision anterior approach to total hip arthroplasty: a multicenter observational study. Orthop Clin North Am. 2009; 40 (3): 329–42.	83	7.84	4
14	Higgins B. T., Barlow D. R., Heagerty N. E., Lin T. J. Anterior vs. posterior approach for total hip arthroplasty, a systematic review and meta-analysis. J Arthroplasty. 2015; 30 (3): 419–34.	81	16.47	12
15	Seng B. E., Berend K. R., Ajluni A. F., Lombardi A. V. Anterior-supine minimally invasive total hip arthroplasty: defining the learning curve. Orthop Clin North Am. 2009; 40 (3): 343–50.	80	7.56	1
16	Goebel S., Steinert A. F., Schillinger J., et al. Reduced postoperative pain in total hip arthroplasty after minimal-invasive anterior approach. Int Orthop. 2012; 36 (3): 491–8.	77	9.73	9
17	Sariali E., Leonard P., Mamoudy P. Dislocation after total hip arthroplasty using hueter anterior approach. J Arthroplasty. 2008; 23 (2): 266–72.	74	6.17	3
18	Zawadsky M. W., Paulus M. C., Murray P. J., Johansen M. A. Early outcome comparison between the direct anterior approach and the mini-incision posterior approach for primary total hip arthroplasty: 150 consecutive cases. J Arthroplasty. 2014; 29 (6): 1256–60.	71	12.53	3
19	Bremer A. K., Kalberer F., Pfirrmann C. W., Dora C. Soft-tissue changes in hip abductor muscles and tendons after total hip replacement: comparison between the direct anterior and the transgluteal approaches. J Bone Joint Surg Br. 2011; 93 (7): 886–9.	71	8.27	7
20	Post Z. D., Orozco F., Diaz-ledezma C., Hozack W. J., Ong A. Direct anterior approach for total hip arthroplasty: indications, technique, and results. J Am Acad Orthop Surg. 2014; 22 (9): 595–603.	68	12.55	22
21	Taunton M. J., Mason J. B., Odum S. M., Springer B. D. Direct anterior total hip arthroplasty yields more rapid voluntary cessation of all walking aids: a prospective, randomized clinical trial. J Arthroplasty. 2014; 29 (9 Suppl): 169–72.	68	12.55	4
22	Christensen C. P., Karthikeyan T., Jacobs C. A. Greater prevalence of wound complications requiring reoperation with direct anterior approach total hip arthroplasty. J Arthroplasty. 2014; 29 (9): 1839–41.	67	12.37	5
23	Kennon R., Keggi J., Zatorski L. E., Keggi K. J. Anterior approach for total hip arthroplasty: beyond the minimally invasive technique. J Bone Joint Surg Am. 2004; 86-A Suppl 2: 91–7.	66	4.15	7
24	De Steiger R. N., Lorimer M., Solomon M. What is the learning curve for the anterior approach for total hip arthroplasty?. Clin Orthop Relat Res. 2015; 473 (12): 3860–6.	60	14.40	7
25	Lovell T. P. Single-incision direct anterior approach for total hip arthroplasty using a standard operating table. J Arthroplasty. 2008; 23 (7 Suppl): 64–8.	60	5.29	3
26	Bhargava T., Goytia R. N., Jones L. C., Hungerford M. W. Lateral femoral cutaneous nerve impairment after direct anterior approach for total hip arthroplasty. Orthopedics. 2010; 33 (7): 472.	58	8.81	1
27	Oinuma K., Eingartner C., Saito Y., Shiratsuchi H. Total hip arthroplasty by a minimally invasive, direct anterior approach. Oper Orthop Traumatol. 2007; 19 (3): 310–26.	58	4.64	0
28	Martin C. T., Pugely A. J., Gao Y., Clark C. R. A comparison of hospital length of stay and short-term morbidity between the anterior and the posterior approaches to total hip arthroplasty. J Arthroplasty. 2013; 28 (5): 849–54.	57	8.44	7
29	Bender B., Nogler M., Hozack W. J. Direct anterior approach for total hip arthroplasty. Orthop Clin North Am. 2009; 40 (3): 321–8.	55	5.20	8
30	Christensen C. P., Jacobs C. A. Comparison of patient function during the first six weeks after direct anterior or posterior total hip arthroplasty (tha): a randomized study. J Arthroplasty. 2015; 30 (9 Suppl):94–7.	50	11.32	8
31	Rathod P. A., Bhalla S., Deshmukh A. J., Rodriguez J. A. Does fluoroscopy with anterior hip arthroplasty decrease acetabular cup variability compared with a nonguided posterior approach?. Clin Orthop Relat Res. 2014; 472 (6): 1877–85.	49	8.65	2
32	Barton C., Kim P. R. Complications of the direct anterior approach for total hip arthroplasty. Orthop Clin North Am. 2009; 40 (3): 371–5.	49	4.63	9
33	Meermans G., Konan S., Das R., Volpin A., Haddad F. S. The direct anterior approach in total hip arthroplasty: a systematic review of the literature. Bone Joint J. 2017; 99-B (6): 732–740.	47	17.63	10
34	De Geest T., Vansintjan P., De loore G. Direct anterior total hip arthroplasty: complications and early outcome in a series of 300 cases. Acta Orthop Belg. 2013; 79 (2): 166–73.	47	6.88	7
35	Rachbauer F., Kain M. S., Leunig M. The history of the anterior approach to the hip. Orthop Clin North Am. 2009; 40 (3): 311–20.	47	4.44	3
36	Hallert O., Li Y., Brismar H., Lindgren U. The direct anterior approach: initial experience of a minimally invasive technique for total hip arthroplasty. J Orthop Surg Res. 2012; 7: 17.	46	5.87	9
37	Restrepo C., Mortazavi S. M., Brothers J., Parvizi J., Rothman R. H. Hip dislocation: are hip precautions necessary in anterior approaches? Clin Orthop Relat Res. 2011; 469 (2): 417–22.	46	5.11	12
38	Lugade V., Wu A., Jewett B., Collis D., Chou L. S. Gait asymmetry following an anterior and anterolateral approach to total hip arthroplasty. Clin Biomech (Bristol, Avon). 2010; 25 (7): 675–80.	46	4.84	11
39	Keggi K. J., Huo M. H., Zatorski L. E. Anterior approach to total hip replacement: surgical technique and clinical results of our first one thousand cases using non-cemented prostheses. Yale J Biol Med. 1993; 66 (3):243–56.	45	1.68	3
40	Judet J., Judet H. [Anterior approach in total hip arthroplasty]. Presse Med. 1985; 14 (18): 1031–3.	44	1.26	2
41	Lee G. C., Marconi D. Complications following direct anterior hip procedures: costs to both patients and surgeons. J Arthroplasty. 2015; 30 (9 Suppl):98–101.	43	9.74	5
42	Maffiuletti N. A., Impellizzeri F. M., Widler K., et al. Spatiotemporal parameters of gait after total hip replacement: anterior versus posterior approach. Orthop Clin North Am. 2009; 40 (3): 407–15.	43	4.06	6
43	Keene G. S., Parker M. J. Hemiarthroplasty of the hip--the anterior or posterior approach? a comparison of surgical approaches. Injury. 1993; 24 (9): 611–3.	40	1.52	2
44	Amlie E., Havelin L. I., Furnes O., et al. Worse patient-reported outcome after lateral approach than after anterior and posterolateral approach in primary hip arthroplasty. a cross-sectional questionnaire study of 1,476 patients 1–23 years after surgery. Acta Orthop. 2014; 85 (5):463–9.	39	7.31	6
45	Rathod P. A., Orishimo K. F., Kremenic I. J., Deshmukh A. J., Rodriguez J. A. Similar improvement in gait parameters following direct anterior and posterior approach total hip arthroplasty. J Arthroplasty. 2014; 29 (6):1261–4.	39	6.88	13
46	Ropars M., Morandi X., Huten D., Thomazeau H., Berton E., Darnault P. Anatomical study of the lateral femoral cutaneous nerve with special reference to minimally invasive anterior approach for total hip replacement. Surg Radiol Anat. 2009; 31 (3):199–204.	39	3.57	4
47	Nogler M., Krismer M., Hozack W. J., Merritt P., Rachbauer F., Mayr E. A. double offset broach handle for preparation of the femoral cavity in minimally invasive direct anterior total hip arthroplasty. J. Arthroplasty. 2006; 21 (8):1206–8.	35	2.66	2
48	Watts C. D., Houdek M. T., Wagner E. R., Sculco P. K., Chalmers B. P., Taunton M. J. High risk of wound complications following direct anterior total hip arthroplasty in obese patients. J Arthroplasty. 2015; 30 (12):2296–8.	34	8.16	4
49	Mjaaland K. E., Kivle K., Svenningsen S., Pripp A. H., Nordsletten L. Comparison of markers for muscle damage, inflammation, and pain using minimally invasive direct anterior versus direct lateral approach in total hip arthroplasty: a prospective, randomized, controlled trial. J Orthop Res. 2015; 33 (9):1305–10.	34	7.70	9
50	Hamilton W. G., Parks N. L., Huynh C. Comparison of cup alignment, jump distance, and complications in consecutive series of anterior approach and posterior approach total hip arthroplasty. J Arthroplasty. 2015; 30 (11):1959–62.	33	7.76	3

## Data Availability

The data are available at https://clarivate.com/webofsciencegroup/solutions/web-of-science/.
